# Punch Trackers: Correct Recognition Depends on Punch Type and Training Experience

**DOI:** 10.3390/s21092968

**Published:** 2021-04-23

**Authors:** Dan Omcirk, Tomas Vetrovsky, Jan Padecky, Sophie Vanbelle, Jan Malecek, James J. Tufano

**Affiliations:** 1 Faculty of Physical Education and Sport, Charles University, 16252 Prague, Czech Republic; tvetrovsky@ftvs.cuni.cz (T.V.); padecky@ftvs.cuni.cz (J.P.); jmalecek@ftvs.cuni.cz (J.M.); tufano@ftvs.cuni.cz (J.J.T.); 2 Department of Methodology and Statistics, Maastricht University, 6211 LK Maastricht, The Netherlands; sophie.vanbelle@maastrichtuniversity.nl

**Keywords:** validity, boxing, mixed martial arts, kickbox, accelerometer

## Abstract

To determine the ability of different punch trackers (PT) (Corner (CPT), Everlast (EPT), and Hykso (HPT)) to recognize specific punch types (lead and rear straight punches, lead and rear hooks, and lead and rear uppercuts) thrown by trained (TR, n = 10) and untrained punchers (UNTR, n = 11), subjects performed different punch combinations, and PT data were compared to data from video recordings to determine how well each PT recognized the punches that were actually thrown. Descriptive statistics and multilevel modelling were used to analyze the data. The CPT, EPT and HPT detected punches more accurately in TR than UNTR, evidenced by a lower percentage error in TR (*p* = 0.007). The CPT, EPT, and HPT detected straight punches better than uppercuts and hooks, with a lower percentage error for straight punches (*p* < 0.001). The recognition of punches with CPT and HPT depended on punch order, with earlier punches in a sequence recognized better. The same may or may not have occurred with EPT, but EPT does not allow for data to be exported, meaning the order of individual punches could not be analyzed. The CPT and HPT both seem to be viable options for tracking punch count and punch type in TR and UNTR.

## 1. Introduction

Boxing is not only a popular combat sport with a long tradition, but it has recently become a popular fitness trend as well [1], with everyday people participating in boxing-related fitness classes hoping to improve their aerobic capacity, reduce their body fat percentage, etc. As this type of training reduces obesity [2], increases cardiovascular health [2,3], and improves aerobic capacity [4], it is no surprise that people seek to participate in this type of high-intensity training. However, as with most types of training, the volume and intensity of training are two of the main factors to consider when designing and implementing a training program. In traditional exercises, such a strength training, it is easy to prescribe a set number of repetitions with a specific load. However, the non-structured, repetitive, and highly dynamic nature of punching makes it difficult to prescribe or quantify training loads. Therefore, it would be advantageous to use technology that could provide objective data to quantify training volume while punching.

Among the available technology today, accelerometers can be used to detect punch type and provide data regarding punch force, velocity, power, and other measures that can help quantify punching training structure, volume, and intensity [5,6,7]. Other technologies available include high-frame-rate video capture [8]. Although accelerometers have been tested during punching [7,9], they have primarily included custom-made devices and algorithms that likely are not used by commercial users. Furthermore, the data collected in those studies are unique to specific audiences (e.g., for scoring and judging strikes, wrist angles, and other variables that everyday users likely are not interested in). Additionally, accelerometers that are invented specifically for the purpose of collecting punching data provide post-workout summaries, known as punch trackers, can even provide instantaneous feedback [9,10], which has been shown to play a role in maximizing acute performance [11] and increasing motivation [12,13,14]. Although these devices are interesting, and the data they provide could be useful, there is a lack of published data to support their validity, likely due to the novelty of the devices.

Although not publicly available, it can be assumed that the algorithms of these punch trackers slightly differ between manufacturers [15]. Furthermore, since the resultant data are based on accelerometry, it is possible that punches thrown with slightly different techniques or trajectories may not be recognized by the punch trackers, reducing their accuracy in terms of quantifying training volume or providing objective feedback. Along these lines, it is possible that the same punches thrown by untrained punchers with less technically correct movement may not be recognized as well as in trained punchers who have better and possibly more consistent punch techniques, especially for more complex movements that require greater coordination (e.g., hooks versus jabs) [16]. Although consumers use these punch trackers during training, their validity has not been assessed in an independent laboratory, which could provide additional information in terms of their ability to function well in real-world settings. Therefore, the purpose of this study was to compare four commercially available punch trackers to determine how well they could recognize specific types of boxing punches thrown by trained punchers and untrained punchers during shadow boxing. This study hypothesized that (I) the punch trackers would better register the total number of punches thrown by trained punchers compared to untrained punchers; (II) simple punches (lead and rear straights) would be detected with higher accuracy than more complex punches, such as lead and rear hooks and uppercuts; and (III) punch recognition would decrease throughout a consecutive sequence due to the hands not “resetting” after each punch, which may not align with the punch algorithms within the devices.

## 2. Methods

### 2.1. Subjects

Twenty-one healthy males, including 10 trained punchers (TR) (28.1 ± 5.5 y, 83 ± 11 kg, 178.2 ± 9.2 cm) and 11 untrained punchers (UNTR) (27.3 ± 6.1 y, 84.5 ± 12.5 kg, 182.6 ± 7.4 cm) volunteered for this study. The TR participants had been formally taught how to execute different types of punches, were experienced with combat sports involving punching for at least one year, and had completed at least one competition fight in any discipline that involved punching (e.g., boxing, mixed martial arts, and kickboxing). The UNTR participants had never been formally taught how to execute different punch techniques and had not participated in any formal fights. All participants had no recent injuries that would affect or be exacerbated by shadow boxing and were allowed to adopt their preferred stance (orthodox or southpaw). All participants provided written informed consent for the study protocols (approval 127/2019).

### 2.2. Design

Participants reported to the laboratory, and all data were collected during a single session. During this session, each participant was familiarized with the testing procedures and completed a standardized series of shadow boxing combinations (i.e., punching the air) with four commercially available punch trackers. All punches were recorded with a video camera (the recordings of which were considered as the gold-standard for punch recognition), and the number of each punch type that actually occurred was later compared to the number of punches provided by each of the punch trackers. In addition to assessing the validity of the punch trackers to recognize the correct punch types in all of the participants, a sub-group analysis compared TR and UNTR to determine if training status, and an assumed better technique in TR, affected the validity of the punch trackers.

### 2.3. Methodology

#### 2.3.1. Warm-Up and Familiarization

Following a standard dynamic warm-up, each participant was provided with the same verbal and physical instructions for each punch type and performed 3 min of technique practice. During this time, the participant stood behind the researcher and performed the same punches as modelled by the researcher (i.e., in the same third-person point of view as the video instructions during the experimental period, explained below). The participants received feedback if the punch was performed incorrectly, and were instructed on how they should adjust their technique so that the punch would be correctly executed. This level of instruction is similar to what a beginner might receive in a group exercise class, increasing the ecological validity of the testing procedures.

#### 2.3.2. Validation Testing

**Figure 1:**
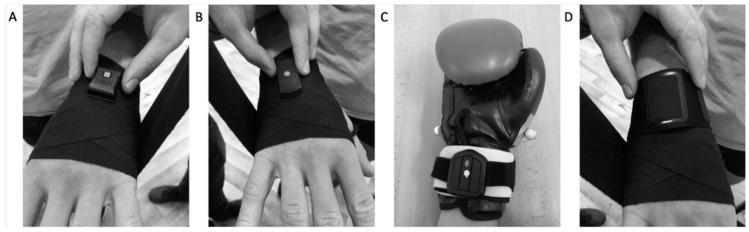
Punch tracker placement: (**A**) the StrikeTec punch tracker inserted directly on top of the wrist, under 2.5 m hand wraps, and under the gloves; (**B**) the Hykso punch tracker inserted directly on top of the wrist, under 2.5 m hand wraps, and under the boxing gloves; (**C**) the Everlast punch tracker attached on the wrist on the surface of the wrist flexors on boxing gloves in their respective wristbands; and (**D**) the Corner punch tracker inserted directly on top of the wrist, under 2.5 m hand wraps, and under gloves, in their respective wristbands.

All testing was performed in the same laboratory with standard 10-ounce boxing gloves and 2.5-m boxing hand wraps that were used to secure the accelerometers according to each manufacturer’s guidelines. The four commercially available punch trackers included models manufactured by Corner (Corner Boxing Trackers, Corner Wearables Ltd., Manchester, UK, v1.3.1 (CPT)), Everlast (Boxing-Sensor System—PiQ Robot^TM^Blue, Everlast Worldwide Inc., Moberly, MO, USA, v2.4.1 (EPT)), Hykso (Hykso Wearable Punch Trackers, Hykso Inc., Costa Mesa, CA, USA., v1.6 (HPT)), and StrikeTec (StrikeTec Boxing Sensors, StrikeTec, Dallas, TX, USA, v1.4.4 (SPT)). The CPT, HPT, and SPT were attached on the wrist on the surface of the wrist extensors, and the EPT was attached on the wrist on the surface of the wrist flexors. The HPT and SPT were inserted directly on top of the wrist, under the 2.5 m hand wraps, and under the gloves, while the CPT and EPT were inserted into their respective wristbands that were sold with the accelerometers; the CPT was then covered by the 2.5 m hand wraps and gloves, and the EPT was attached on the wrist (Figure 1). Each punch tracker was used as a pair, attached to the lead and rear hand.

Since some of the punch trackers have the same recommended placement, they were not all used at the same time, resulting in three separate but identical rounds of shadow boxing. To avoid any potential order effect, the order of the accelerometers was randomized in a counter-balanced fashion among the participants. Each round of shadow boxing included a standard set of 54 punches that included lead straight punches (LS), rear straight punches (RS), lead hooks (LH), rear hooks (RH), lead uppercuts (LUC), and rear uppercuts (RUC). To avoid any order effect for punch type within any possible punch combination, the punches were split into series that included a pyramid of six single-punches, six double-punch sequences, six triple-punch sequences, six double-punch sequences, and six single-punches (54 total punches per round). The sequences of punch combinations were randomized, but the number of punch types per sequence was constant for every participant, and every participant performed the same set of punches in the same sequence (see Table 1 for an example).

**Figure 2:**
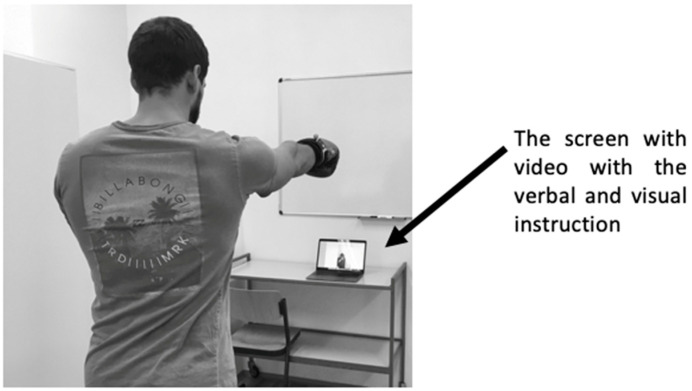
Instructional video: the participant stood in front of the screen with the video with verbal and visual instructions.

Participants performed all of the punches as fast and hard as possible (with maximal effort). Before each punch series, each participant was shown an identical video with the same verbal and visual instructions. The video was shown on a laptop and included a member of the research team performing the upcoming punches using a third-person view from the rear, as this set-up seemed best for the UNTR to mimic during pilot testing (Figure 2). There was 10 s of rest between each combination.

After completing a round of 54 punches, 5 min of rest was provided, and the next punch tracker was placed on the participant. The same procedures occurred for the second round (i.e., second punch tracker), followed by 5 min of rest, and then the final round. The order of PTs was randomized for each round, but since the EPT was attached on the opposite side of the wrist, it was randomly placed during the first, second, or final round, meaning that one of the three rounds included the EPT and one of the other punch trackers simultaneously.

#### 2.3.3. Data Acquisition

All punches were video-recorded on a tablet from the rear at a 45-degree angle, allowing the main investigator to clearly analyze the exact number of punches for each type. Mainly in the UNTR group, it was possible that a participant accidentally threw the wrong type of punch in a specific series. For example, instead of performing an RH, they performed an RUC. In these cases, the actual punch type that was thrown (assessed via video) was recorded, as that was the punch type to be recognized by the punch tracker. Data from CPT and HPT were transmitted via Bluetooth to a laptop, and their respective data were exported in a csv file and converted to Microsoft Excel for future analysis. Data from EPT and SPT were rewritten from their respective mobile applications (EPT—Everlast and PIQ; SPT—StrikeTec Boxing), because they do not allow for direct export to a csv file. Thus, the data were manually imported to Microsoft Excel. Due to technical failures and incomplete/missing data sets for some participants, data from all 21 participants were not always included in the final analyses. Therefore, the final participant counts with full data sets were as follows: HPT (n = 21); CPT (n = 20); EPT (n = 18); and SPT (n = 0). The information provided by each punch tracker is shown in Table 2. As a note, the SPT would only register a few “random” punches for a select few participants (a mixture of TR and UNTR). Therefore, it is possible that the devices were faulty, or that they were not operated correctly, but it is also possible that the SPT simply did not work as expected. It is not known exactly what the problem was, but future research should determine the efficacy of SPT and whether another set of SPT performs similarly.

#### 2.3.4. Statistical Analyses

To assess the validity of the punch trackers to determine the total punch count during shadow boxing, percentage errors between the recorded (by the tracker) and true (as determined from video recording) number of punches were calculated for each round of shadow boxing. Based on this, mean percentage errors (MPE) and mean absolute percentage errors (MAPE) with their 95% confidence intervals were calculated for all participants combined and separately for TR and UNTR subgroups. Furthermore, equivalence testing was carried out by the two one-sided tests (TOST) method with α = 0.05. The equivalence zone was defined as within ±10% of the true punch count. To assess the effect of training and of punch type on the log-transformed percentage errors, linear mixed effect models were fitted using lme4 (version 1.1-20) and lmerTest (version 3.1-0) packages in R, version 3.5.2 (The R Foundation for Statistical Computing, Vienna, Austria). To determine the effect of specific punch types on the log-transformed percentage errors, post hoc pairwise comparison using general linear hypotheses testing was performed with the multcomp (version 1.4-8) package in R. 

To assess the validity of the punch trackers to recognize individual punch types, sensitivity and specificity were calculated for each punch type. Sensitivity was calculated as the ratio of correctly recognized (true positive) punches and all punches of a given type; specificity was calculated as the ratio of punches correctly recognized as not being of a given type (true negative) and all punches not being of a given type. Furthermore, logistic regression with mixed effects was used to assess the effect of context (i.e., order of the punch within a sequence, early vs. late in the round) on the ability of the punch trackers to correctly identify and recognize individual punches. The data presented in this study are available in supplementary materials.

## 3. Results

The total punch counts for each punch type and each punch tracker are shown in Table 3. The punches in the video recordings were all able to be identified as a specific punch type by the researcher, indicating that the movement pattern of the subject’s hands matched what would be expected for such a punch type. Therefore, the researcher judged that all punches were performed within the expected movement patterns, but it was possible that subjects performed an LH instead of an LUC (for example). In these cases, the LH was the actual punch thrown, which was recognized by the punch tracker. The results of MPE, MAPE and TOST for CPT, EPT and HPT for all participants and each group (TR and UNTR) are shown in Table 4. The linear mixed-effects model indicated that the percentage error was significantly affected by punch type (*p* < 0.001) and training experience (*p* = 0.007). Specifically, the post hoc analysis revealed that the percentage error was lower for straight punches (lead and rear) compared to hooks and uppercuts (*p* < 0.001) for all three punch trackers (Table 5).

The sensitivity and specificity for CPT and HPT for recognizing individual punches (LS, RS, LH, RH, LUC and RUC) are present in Table 6. The logistic regression with mixed effects indicated that there was a significant negative effect of the order within a sequence (*p* < 0.001 for CPT and *p* < 0.001 for HPT) and positive effect of the position within a round (*p* = 0.024 for CPT and *p* = 0.003 for HPT). In other words, the earlier within a sequence and the later within a round the punch was thrown, the better it was recognized.

Using the straight lead as an example, sensitivity is the proportion of straight lead punches that were correctly recognized as such, and specificity is the proportion of non-straight lead punches that are recognized as non-straight lead punches (but not necessarily recognized correctly). Sensitivity and specificity values as close as possible to one are desired.

## 4. Discussion

The main findings are that (I) the CPT, EPT, and HPT all detected punches with more accuracy in TR than UNTR participants; (II) the CPT, EPT, and HPT were all better at detecting straight punches compared to uppercuts and hooks; and (III) the successful recognition of punches with CPT and HPT depended on the order of boxing punches, with earlier punches in a sequence being recognized better. The same may or may not have occurred with the EPT, but the device does not allow for data to be exported, meaning individual punch data, such as the order of individual punches, could not be analyzed.

Based on the data presented, which supported the first hypothesis, participants with combat sport experience can use CPT, EPT and HPT to detect the total number of punches per session with reasonable accuracy. However, in UNTR participants, the EPT and HPT underestimated the total punch count, meaning that the CPT may be a better choice for untrained punchers in this regard. Considering that the punch trackers used in this study likely have unique algorithms for identifying different punch types [1,15] the technical implementation of each punch likely played a major role in the ability of each punch tracker to correctly register every punch. Since the EPT and HPT underestimated the total punch count in UNTR, it is possible that the thresholds needed to register a punch were not met, which could be a result of greater variability in the punch technique in UNTR compared to TR [16].

Considering the punch technique, the second hypothesis was also confirmed as the CPT, EPT, and HPT were all able to better detect straight punches than hooks and uppercuts (Table 5). Specifically, the HPT had better sensitivity (recognition) than CPT for straight punches. However, the CPT was better than the HPT for correctly detecting hooks and uppercuts. Since hooks and uppercuts are delivered in a curved swinging motion with a vertical drop in the initiation of the punch, they are more technical and complex than straight punches [16]. Therefore, the UNTR punchers likely were unable to maintain the proper technique, resulting in worse upper cut and hook detection by the punch trackers compared to TR. Considering the strict technical requirements of hooks and uppercuts compared to straight punches, the likelihood of a “false-positive” decreases for hooks and upper cuts, which is supported by a greater specificity for hooks and uppercuts than straight punches (Table 6). In short, if a punch tracker registered a hook or uppercut, it likely actually was a hook or uppercut, since a straight punch would likely not include an arcing pattern, even for the most inexperienced punchers.

The third hypothesis was also confirmed because regardless of training experience, increasing the number of punches in a sequence negatively influenced the recognition of punch type as the order of punches progressed. Although it is possible that the participants were able to focus better on the first punch of a multipunch sequence, losing their focus as the sequence progressed, the more likely explanation is that the first punch was performed from a static position. For subsequent punches, the punch trackers may not have registered returning to the start position, which may reduce their ability to correctly detect the next punch. Furthermore, the technique of transitioning from one punch to the next simply may not have corresponded with the movements that were expected in the respective algorithms. Contrary to the negative effect of the order of punches within a sequence, as each round of shadow boxing progressed (i.e., after multiple sequences), the CPT and HPT better recognized punch types in both TR and UNTR participants. It is possible that there was a learning effect, which has previously been shown to increase punch force and velocity after only 15 min of practice [17], but such rapid skill acquisition would have likely occurred only in UNTR. Nevertheless, the present data do not allow for such a conclusion, and the most logical explanation for the increased recognition over time is the pyramid nature of the protocol. Subjects performed a series of single punches, followed by two-punch combinations, three-punch combinations, two-punch combinations, and finished with single punches. As such, the latter punches of the round were in fact single punches, meaning that the number of punches per sequence likely plays a greater role in punch recognition than the overall time spent punching. Therefore, any possible learning effect may be negligible in such a short time period, and the transitions between punches (i.e., the lack of coming back to a static starting position) likely make it difficult for the punch trackers to correctly identify multiple punches in sequence. 

In addition to the main findings above, there are many factors to consider when interpreting the data of the present study. First, the maximum number of punches in a sequence was three. Considering the negative effect of the number of punches in a sequence on proper recognition, the data from each punch tracker would likely differ, and possibly worsen, if the number of punches per sequence increased past three. Thus, future research should investigate the punch recognition ability of these trackers in situations where many punches are performed in sequence. Second, the EPT only provides average data from the whole session for each punch type (Table 2), meaning that punch-by-punch analyses are not possible, which is a factor to consider depending on the user’s needs. Third, due to an insufficient amount of data (Table 3), the SPT data were not analyzed. Therefore, it cannot be concluded that SPT is not reliable for detecting punch types, as the SPT used in the present study may have been defective. On the other hand, it may not have been defective, and future research should aim to determine how the SPT performs under different conditions. Fourth, the CPT and HPT likely provide the most valid data for detecting and recognizing punch types. For detecting the total punch count, the CPT and HPT are both acceptable, particularly the CPT for participants without any training experience, and the HPT for more experienced participants. Although CPT, HPT, and EPT were better at detecting straight punches than hooks and uppercuts, a punch-by-punch analysis showed that the CPT and HPT not only detected but successfully recognized straight punches better than hooks and uppercuts (the EPT does not allow for such an analysis). 

The CPT and HPT can both be used to evaluate shadow boxing with multiple punches, but single punches would likely be recognized more accurately. Lastly, the protocols were performed under standardized conditions, with a specific count of punches and combinations, all while boxing without an opponent. Thus, altering any combination of these conditions may affect the ability of these punch trackers to provide valid punch data, and future research should investigate these effects.

## 5. Practical Applications

The CPT and HPT likely provide the most valid data in terms of detecting and recognizing punch types and the total punch count during shadow boxing. Specifically, the CPT may be more suitable for participants without much experience, and the HPT may be more suitable for experienced punchers. 

## 6. Conclusions

The findings can help potential users of punch trackers choose a device based on their preferences, the possibility of exporting individual punch data, their level of experience, and the ability to detect and recognize different punch types. Nevertheless, it is important that future research investigates the punch recognition abilities of these punch trackers in other scenarios where large numbers of punches are performed in sequence, which based on the current findings, would likely reduce the accuracy of the data.

## Figures and Tables

**Table 1:** An example of 1 round of shadow boxing.

Order	Single	Order	Double	Order	Triple	Order	Double	Order	Single
1	LS	7–8	LS+RS	19–21	LS+RS+LH	37–38	LH+RS	49	RS
2	RS	9–10	RS+LH	22–24	RS+LS+RUC	39–40	RUC+LUC	50	LUC
3	RH	11–12	RH+LUC	25–27	LUC+RH+LS	41–42	LS+RH	51	LH
4	LH	13–14	RUC+LS	28–30	RH+LH+RH	43–44	RUC+LH	52	LS
5	LUC	15–16	LUC+RH	31–33	LH+RUC+LUC	45–46	RH+RUC	53	RUC
6	RUC	17–18	LH+RUC	34–36	RUC+LUC+RS	47–48	RS+LS	54	RH

Lead straight punch (LS), rear straight punch (RS), lead hook (LH), rear hook (RH), lead uppercut (LUC), and rear uppercut (RUC).

**Table 2:** Information provided by each punch tracker (PT).

PT	PT Placement	Manufacturer’s Wraps	Strike Speed	Intensity/Power Output	Strike Count	Strike Type	Export Function *
Hykso	Wrist (inside glove)	No	Yes (maximum)	Intensity score	Yes	Yes	Yes
Corner	Wrist (inside glove)	Yes	Yes (unspecified)	Power G	Yes	Yes	Yes
StrikeTec	Wrist (inside glove)	No	Yes (unspecified)	Power (LBS/F)	Yes	Yes	No
Everlast PIQ	Wrist (outside glove)	Yes	Yes (average)	G-Force, avg. PIQScore, max. retraction	Yes	Yes	No

* The StrikeTec shows individual punch data, but they cannot be exported for external use. The Everlast PIQ neither shows the data for individual punches nor allows the workout summary to be exported for external use. Other information, such as the sampling frequency, is not provided, and the companies did not respond to the request for any extra information.

**Table 3:** Mean and standard deviation of shadow boxing punches that were recorded by each punch tracker and the actual punches that were thrown.

	HPT (TR, n = 10; UNTR, n = 11)		CPT (TR, n = 10; UNTR, n = 10)		EPT (TR, n = 7; UNTR, n = 11)		SPT (TR, n = 6; UNTR, n = 11)	
	Tracker	Actual	Tracker	Actual	Tracker	Actual	Tracker	Actual
Total TR	53.2 ± 2.8	53.9 ± 0.3	55.6 ± 4.8	53.9 ± 0.3	57.3 ± 8.5	54.0 ± 0.0	12.7 ± 8.0	54.0 ± 0.0
Total UNTR	46.5 ± 7.4	54.0 ± 0.0	52.7 ± 2.2	53.8 ± 0.6	45.8 ± 7.0	54.0 ± 0.3	9.6 ± 10.3	54.0 ± 0.0
LS TR	12.5 ± 2.0	9.2 ± 0.6	11.1 ± 2.8	9.1 ± 0.3	11.7 ± 5.1	9.3 ± 0.7	5.3 ± 2.4	9.0 ± 0.0
LS UNTR	10.6 ± 2.1	8.8 ± 0.7	12.0 ± 4.1	8.9 ± 0.3	12.5 ± 4.9	8.9 ± 0.5	3.7 ± 3.9	9.0 ± 0.4
RS TR	13.5 ± 4.5	8.8 ± 0.6	13.3 ± 4.1	8.9 ± 0.3	10.4 ± 2.5	8.7 ± 0.7	5.5 ± 4.2	9.0 ± 0.0
RS UNTR	10.5 ± 1.9	9.1 ± 0.3	11.7 ± 2.9	9.1 ± 0.3	9.6 ± 1.8	9.2 ± 0.6	5.8 ± 6.7	9.0 ± 0.4
LH TR	9.1 ± 2.5	9.4 ± 0.7	8.6 ± 3.4	8.8 ± 0.6	8.0 ± 2.7	9.3 ± 0.5	0.5 ± 0.8	9.2 ± 0.4
LH UNTR	4.9 ± 2.1	9.1 ± 0.3	7.0 ± 3.7	9.3 ± 1.2	4.2 ± 3.9	9.6 ± 1.2	0.1 ± 0.3	9.5 ± 1.2
RH TR	6.4 ± 1.7	8.8 ± 0.4	5.3 ± 3.4	9.1 ± 0.8	8.3 ± 7.7	8.9 ± 0.4	1.3 ± 2.0	8.8 ± 0.4
RH UNTR	5.9 ± 2.7	9.4 ± 0.8	4.4 ± 2.5	9.0 ± 0.4	8.6 ± 3.1	8.9 ± 0.8	0.0 ± 0.0	8.7 ± 0.6
LUC TR	4.7 ± 2.1	8.0 ± 0.6	7.0 ± 1.7	7.9 ± 0.5	6.7 ± 3.3	7.7 ± 0.5	0.0 ± 0.0	7.8 ± 0.4
LUC UNTR	6.0 ± 2.9	8.3 ± 0.8	6.9 ± 2.8	8.2 ± 0.7	5.9 ± 3.5	8.5 ± 1.2	0.0 ± 0.0	8.5 ± 1.2
RUC TR	7.0 ± 3.6	9.7 ± 0.6	9.3 ± 4.0	10.2 ± 0.8	9.7 ± 5.0	10.1 ± 0.4	0.0 ± 0.0	10.2 ± 0.4
RUC UNTR	8.6 ± 1.2	9.4 ± 0.6	9.9 ± 2.6	9.4 ± 1.2	5.2 ± 3.8	9.1 ± 1.5	0.0 ± 0.0	9.3 ± 1.5

Hykso (HPT), Corner (CPT), Everlast (EPT), and StrikeTec (SPT) and the actual number of punches thrown for trained participants (TR) and trained participants (UNTR). The standard set for each punch tracker (TR and UNTR) consisted of 54 punches; lead straight (LS (n = 9)), rear straight (RS (n = 9)), lead hook (LH (n = 9)), rear hook (RH (n = 9)), lead uppercut (LUC (n = 8)), and rear uppercut (RUC (n = 10)). For example, if a subject was supposed to perform an RH, RS, RUC, but they instead performed RH, RS, RH, the “RH, RS, RH” is what was actually thrown, so that should have been what the punch trackers recognized.

**Table 4:** Summary results for each punch tracker across all participants, trained participants, and untrained participants.

	MPE (95% Confidence Limits)	MAPE (95% Confidence Limits)	TOST *p*-Value
All participants			
CORNER	0.005 (−0.080 to 0.090)	0.031 (0.000 to 0.207)	0.014
EVERLAST	−0.058 (−0.159 to 0.044)	0.127 (0.000 to 0.405)	0.208
HYKSO	−0.080 (−0.153 to −0.007)	0.095 (0.000 to 0.343)	0.296
Trained participants			
CORNER	0.031 (−0.096 to 0.158)	0.043 (0.000 to 0.236)	0.142
EVERLAST	0.065 (−0.093 to 0.223)	0.066 (0.000 to 0.381)	0.332
HYKSO	−0.016 (−0.136 to 0.104)	0.043 (0.000 to 0.090)	0.086
Untrained participants			
CORNER	−0.020 (−0.134 to 0.093)	0.020 (0.000 to 0.113)	0.084
EVERLAST	−0.136 (−0.266 to −0.006)	0.165 (0.005 to 0.338)	0.706
HYKSO	−0.138 (−0.023 to −0.054)	0.143 (0.000 to 0.347)	0.813

Mean percentage error (MPE) and mean absolute percentage error (MAPE) with their 95% confidence limits, and equivalence test (TOST *p*-value), are shown. The advisable values for MPE and MAPE are close to zero.

**Table 5:** Pairwise comparison of the effect of the punch type.

Punch Types Compared	ß	*p*-Value	Punch Types Compared	ß	*p*-Value	Punch Types Compared	ß	*p*-Value
LS-RS	−0.000	1	LH-LS	−0.270	<0.001	RH-LH	−0.045	0.859
LH-RS	−0.270	<0.001	LUC-LS	−0.259	<0.001	RUC-LH	0.049	0.805
LUC-RS	−0.260	<0.001	RH-LS	−0.315	<0.001	RH-LUC	−0.056	0.710
RH-RS	−0.315	<0.001	RUC-LS	−0.221	<0.001	RUC-LUC	0.038	0.922
RUC-RS	−0.221	<0.001	LUC-LH	0.011	1	RUC-RH	0.094	0.151

Lead straight (LS), rear straight (RS), lead hook (LH), rear hook (RH), lead uppercut (LUC) and rear uppercut (RUC) on percentage error of the three punch trackers combined (Corner, Hykso, Everlast). ß expresses a difference between percentage errors achieved by the two punch types. For example, in the second row (LH-RS), ß-value of −0.270 means that the percentage error achieved by RS is lower by 0.270 compared to the percentage error achieved by LH.

**Table 6:** Sensitivity and specificity of Corner and Hykso punch trackers to correctly recognize individual punches.

	Corner		Hykso	
Punch	Sensitivity	Specificity	Sensitivity	Specificity
STRAIGHT LEAD	0.833	0.917	0.958	0.935
STRAIGHT REAR	0.911	0.895	0.947	0.927
LEAD HOOK	0.648	0.958	0.521	0.958
REAR HOOK	0.538	0.991	0.497	0.960
LEAD UPPERCUT	0.741	0.977	0.560	0.976
REAR UPPERCUT	0.783	0.956	0.665	0.972

## Data Availability

The data generated and analyzed during this study are included in this article. Raw data files used to generate figures shown in this work were uploaded as a supplementary file.

## Supplementary Materials

The raw data are available online at https://www.mdpi.com/article/10.3390/s21092968/s1, Table S1: manuscript-supplementary.
