# Supplementary material for: Punch Trackers: Correct Recognition Depends on Punch Type and Training Experience
Source: Sensors (Basel). 2021 Apr 23;21(9):2968. doi: 10.3390/s21092968 (PMC8123076; doi:10.3390/s21092968)
Supplement: Supplementary file 1 [file sensors-21-02968-s001.zip › sensors-1173148-SI.pdf]

SUB	ACC	CAT	REAL	REC
1	HYKSO	A	J	J
			C	C
			RH	0
			LH	0
			LUC	0
			RUC	RH
			J	J
			C	C
			C	C
			LH	J
			RH	RH
			LUC	0
			RUC	RH
			J	J
			LUC	0
			RH	C
			LH	0
			RUC	RH
			J	J
			C	C
			LH	LUC
			C	C
			J	J
			RH	RUC
			LUC	0
			RH	RH
			J	J
			RH	RUC
			LH	J
			RH	C
			LH	LH
			RUC	RUC
			LUC	0
			RUC	RUC
			LUC	0
			C	C
			LH	J
			C	C
			RUC	RUC
			LUC	LUC

			J	J
			RH	RUC
			RUC	RUC
			LH	LH
			RH	RH
			RUC	RUC
			C	C
			J	J
			C	C
			LUC	LUC
			LH	0
			J	J
			RUC	RUC
			RH	RH
1	CORNER	A	J	J
			C	C
			RH	RH
			LH	LH
			LUC	LH
			RUC	RUC
			J	C
			C	J
			C	C
			LH	J
			RH	RH
			LUC	LH
			RUC	RUC
			J	J
			LUC	LH
			RH	RH
			LH	LH
			RUC	RUC
			J	J
			C	C
			LH	LH
			C	C
			J	J
			RH	RUC
			LUC	LH
			RH	RH
			J	J
			RH	RH

			LH	LH
			RH	RH
			LH	LH
			RUC	RUC
			LUC	LH
			RUC	RUC
			LUC	LUC
			C	C
			LH	LH
			C	C
			RUC	RUC
			LUC	LH
			J	J
			RH	RUC
			RUC	RUC
			LH	LH
			RH	RH
			RUC	RUC
			C	J
			J	C
			C	C
			LUC	LUC
			LH	LH
			J	J
			RUC	RUC
			RH	RH
2	HYKSO	A	J	J
			C	C
			RH	0
			LH	LH
			LUC	LUC
			RUC	RUC
			J	J
			C	C
			C	C
			LH	LH
			RH	C
			LUC	LH
			RUC	RUC
			J	J
			LUC	LUC
			RH	RH

			LH	LUC
			RUC	C
			J	J
			C	C
			LH	J
			C	C
			J	J
			RUC	RUC
			LUC	LUC
			RH	C
			J	J
			RH	RH
			LH	LH
			RH	C
			LH	LUC
			RUC	RUC
			LUC	LUC
			RUC	RH
			LUC	LUC
			C	C
			LH	LUC
			C	C
			RUC	RUC
			LUC	LUC
			J	J
			RH	C
			RUC	RUC
			LH	J
			RH	RH
			RUC	C
			C	C
			J	J
			C	C
			LUC	LUC
			LH	LH
			J	J
			RUC	RUC
			RH	RH
3	HYKSO	A	C	C
			J	J
			LH	LH
			RH	0

			RUC	RUC
			LUC	LUC
			C	C
			J	J
			RH	RUC
			LH	0
			LH	LUC
			RUC	C
			LUC	RUC
			C	RUC
			RUC	RUC
			RH	0
			RUC	C
			LH	J
			C	J
			J	C
			RH	J
			J	0
			C	LUC
			RH	0
			RUC	0
			LH	LH
			LUC	0
			LH	0
			RH	0
			LH	J
			RH	LUC
			LUC	RUC
			RH	J
			LUC	0
			RUC	C
			J	0
			RH	LUC
			C	RUC
			LUC	0
			RUC	J
			C	0
			LH	0
			LUC	0
			RH	RH
			LUC	0
			LH	0

			J	J
			C	C
			J	J
			RUC	RUC
			RH	0
			C	0
			LUC	J
			LH	LH
3	CORNER	A	C	C
			J	J
			LH	C
			RH	J
			LUC	LUC
			RUC	RUC
			C	C
			J	J
			J	J
			LH	LH
			LH	LH
			RUC	RUC
			LUC	LUC
			J	J
			LH	C
			RUC	RUC
			RH	J
			LUC	LUC
			C	J
			J	C
			LH	C
			J	J
			C	C
			RH	J
			RUC	RUC
			LH	C
			C	C
			LH	LH
			RH	RH
			LH	LUC
			RH	LUC
			LUC	J
			RUC	RUC
			LH	LH

			RH	J
			J	J
			RH	J
			C	C
			LUC	LUC
			RUC	RUC
			C	C
			LH	LH
			LUC	LUC
			RH	RH
			0	0
			0	0
			J	J
			C	C
			J	J
			LH	C
			LH	C
			C	C
			LUC	LUC
			LH	C
4	HYKSO	F	J	J
			C	C
			RH	RH
			LH	LH
			LUC	LUC
			0	J
			RUC	RUC
			J	J
			C	C
			C	C
			LH	LH
			RH	RH
			LUC	LH
			RUC	RUC
			J	J
			LUC	LUC
			RH	RUC
			LH	LH
			RUC	RH
			J	J
			C	C
			LH	LH

			C	C
			J	J
			RUC	RUC
			LUC	LUC
			RH	RUC
			J	J
			RH	RUC
			LH	LH
			RH	RH
			LH	LH
			RUC	RH
			LUC	J
			RUC	RUC
			LUC	LUC
			C	C
			LH	LH
			C	C
			RUC	RUC
			LUC	LH
			J	J
			RH	RH
			RUC	RUC
			LH	LH
			0	J
			RH	RUC
			RUC	RUC
			C	C
			J	J
			C	C
			LUC	LUC
			LH	LUC
			J	J
			0	J
			RH	RUC
			0	J
			RUC	RUC
4	CORNER	F	J	J
			C	0
			RH	RH
			LH	LH
			LUC	LUC
			RUC	RUC

			J	C
			C	J
			C	C
			LH	LH
			RH	RH
			LUC	LUC
			RUC	RUC
			J	LH
			LUC	RH
			RH	LUC
			LH	RUC
			RUC	LH
			J	LH
			C	J
			LH	C
			C	C
			J	J
			RUC	RH
			LUC	LUC
			RH	RH
			J	LH
			RH	RH
			LH	LH
			RH	RH
			LH	LH
			RUC	RUC
			LUC	LUC
			RUC	RUC
			LUC	LUC
			C	C
			LH	LH
			C	C
			RUC	RUC
			LUC	LUC
			J	J
			RH	RH
			RUC	RUC
			LH	LH
			RH	RH
			RUC	RUC
			C	C
			J	J

			C	C
			LUC	LUC
			LH	LH
			J	J
			RUC	RUC
			RH	RH
5	HYKSO	A	J	J
			C	C
			RH	RUC
			LH	0
			LUC	LUC
			RUC	RH
			J	J
			C	C
			C	C
			LH	0
			RH	RH
			LUC	LUC
			RUC	RUC
			J	J
			LUC	LUC
			RH	RUC
			LH	LH
			RUC	RUC
			J	J
			C	C
			LH	0
			C	C
			J	0
			RUC	RUC
			LUC	LUC
			RH	RUC
			J	J
			RH	0
			LH	J
			RH	RH
			LH	0
			RUC	RUC
			LUC	LH
			RUC	RUC
			LUC	LUC
			C	C

			LH	LH
			C	C
			RUC	RUC
			LUC	LUC
			J	J
			RH	RH
			RUC	0
			LH	LH
			RH	RUC
			RUC	RH
			C	C
			J	J
			J	J
			LUC	LUC
			LH	0
			C	C
			RUC	0
			RH	RUC
5	CORNER	A	J	J
			C	C
			RH	RH
			LH	LH
			LUC	LUC
			RUC	RUC
			J	J
			C	C
			C	C
			LH	LH
			RH	RUC
			LUC	LUC
			RUC	RUC
			J	0
			LUC	LUC
			RH	RUC
			LH	LH
			RUC	0
			J	J
			C	C
			LH	LH
			C	C
			J	J
			RUC	0

			LUC	LUC
			RH	0
			J	J
			RH	RH
			LH	J
			RH	0
			LH	LH
			RUC	RUC
			LUC	LUC
			RUC	0
			LUC	LUC
			C	C
			LH	LH
			C	C
			RUC	RUC
			LUC	LUC
			J	J
			RH	RH
			RUC	RUC
			LH	LH
			RH	RH
			RUC	RUC
			C	C
			J	J
			C	C
			LUC	LUC
			LH	LH
			J	0
			RUC	RUC
			RH	RUC
6	HYKSO	A	J	J
			C	C
			RH	RH
			LH	LH
			LUC	LUC
			RUC	RUC
			J	LH
			C	C
			C	C
			LH	LH
			RH	RH
			LUC	LH

			RUC	RUC
			J	J
			LUC	LUC
			RH	RH
			LH	LH
			RUC	RUC
			J	J
			C	C
			LH	LH
			C	C
			J	J
			RUC	RH
			LUC	LUC
			RH	RH
			J	J
			RH	RH
			LH	LH
			RH	C
			LH	J
			RUC	RUC
			LUC	LUC
			RUC	RUC
			LUC	LH
			0	J
			C	C
			LH	LH
			C	C
			RUC	RUC
			LUC	LUC
			J	J
			RH	RH
			RUC	RUC
			LH	LUC
			RH	RH
			RUC	RUC
			C	C
			J	J
			C	C
			LUC	LUC
			LH	LH
			J	J
			RUC	RUC

			RH	RH
6	CORNER	A	J	J
			C	C
			RH	RUC
			LH	LUC
			LUC	LUC
			RUC	RUC
			J	J
			C	C
			C	C
			LH	J
			RH	RUC
			LUC	LUC
			RUC	RUC
			J	J
			LUC	RUC
			RH	LUC
			LH	LH
			RUC	RUC
			J	J
			C	LH
			LH	C
			C	J
			J	C
			RUC	RUC
			LUC	LUC
			RH	RH
			J	0
			RH	RH
			LH	J
			RH	LH
			LH	LH
			RUC	RUC
			LUC	LUC
			RUC	RUC
			LUC	LUC
			C	C
			LH	C
			C	LH
			RUC	RUC
			LUC	LH
			J	J

			RH	RH
			RUC	LH
			LH	RUC
			RH	RH
			RUC	RUC
			C	C
			J	J
			C	C
			LUC	LH
			LH	LH
			C	C
			RUC	RUC
			RH	RH
7	HYKSO	A	C	C
			J	J
			LH	LH
			RH	J
			RUC	RUC
			LUC	LH
			C	C
			J	J
			J	J
			RH	J
			LH	C
			RUC	RUC
			LUC	LUC
			J	J
			RUC	RUC
			LH	LUC
			RH	RH
			LUC	LUC
			C	C
			J	J
			RH	RH
			J	J
			C	C
			LUC	LH
			RUC	RUC
			LH	LH
			C	C
			LH	LH
			RUC	RUC

			**LH**	**C**
			**RH**	**RH**
			**LUC**	**LUC**
			**RUC**	**RUC**
			**LUC**	**LUC**
			**RUC**	**RUC**
			**J**	**J**
			**RH**	**RH**
			**C**	**C**
			**LUC**	**LUC**
			**RUC**	**RUC**
			**C**	**C**
			**LH**	**C**
			**LUC**	**LH**
			**RH**	**RUC**
			**LH**	**C**
			**LUC**	**LUC**
			**J**	**J**
			**C**	**C**
			**J**	**J**
			**RUC**	**RUC**
			**RH**	**J**
			**C**	**C**
			**LUC**	**LUC**
			**LH**	**LUC**
7	CORNER	A	**C**	**C**
			**J**	**J**
			**LH**	**LH**
			**RH**	**RH**
			**RUC**	**RUC**
			**LUC**	**LUC**
			**C**	**J**
			**J**	**C**
			**J**	**J**
			**RH**	**RUC**
			**LH**	**RUC**
			**RUC**	**LH**
			**LUC**	**LUC**
			**C**	**C**
			**RUC**	**RUC**
			**LH**	**LH**
			**RH**	**RH**

			LUC	LUC
			C	C
			J	J
			RH	RUC
			J	J
			C	C
			LUC	LUC
			RUC	RUC
			LH	LUC
			C	C
			LH	LH
			RH	LH
			LH	RUC
			RH	RH
			LUC	LUC
			RUC	RUC
			LUC	LH
			RUC	RUC
			J	J
			RH	RH
			J	J
			LUC	LUC
			RUC	RUC
			C	C
			LH	C
			LUC	LH
			RH	RUC
			LH	LH
			LUC	LUC
			J	J
			C	C
			J	J
			LUC	LUC
			RH	RH
			C	C
			RUC	RUC
			LH	LH
8	HYKSO	F	J	J
			C	C
			RH	C
			LH	J
			LUC	LUC

			RUC	RH
			J	J
			C	C
			C	C
			LH	LUC
			RH	C
			LUC	LUC
			RUC	RUC
			J	J
			LUC	LUC
			RH	C
			LH	J
			RUC	RH
			J	J
			C	C
			LH	J
			C	C
			J	J
			RUC	RUC
			LUC	0
			RH	C
			J	J
			RH	RH
			LH	J
			RH	C
			LH	0
			RUC	RH
			LUC	LUC
			RUC	RUC
			LUC	0
			C	C
			LH	LH
			C	C
			RUC	RUC
			LUC	LUC
			J	J
			RH	C
			RUC	RUC
			LH	LH
			RH	RH
			RUC	RUC
			C	C

			J	J
			C	C
			LUC	LUC
			LH	LH
			J	J
			RUC	RUC
			RH	RH
8	CORNER	F	J	J
			C	C
			RH	RH
			LH	J
			LUC	LUC
			RUC	RUC
			J	J
			C	C
			C	C
			LH	J
			RH	RH
			LUC	LUC
			RUC	RUC
			J	J
			LUC	LUC
			RH	RH
			LH	J
			RUC	RUC
			J	J
			C	C
			LH	LH
			C	C
			J	J
			RUC	RUC
			LUC	LUC
			RH	C
			J	J
			RUC	RUC
			LUC	LUC
			RUC	RUC
			LH	J
			RUC	RUC
			LUC	LUC
			RUC	RUC
			LUC	LUC

			**C**	**C**
			**LH**	**J**
			**C**	**C**
			**RUC**	**RUC**
			**LUC**	**LUC**
			**J**	**J**
			**RH**	**C**
			**RUC**	**RUC**
			**LH**	**LH**
			**RH**	**RH**
			**RUC**	**RUC**
			**J**	**J**
			**C**	**C**
			**C**	**C**
			**LUC**	**LUC**
			**LH**	**J**
			**J**	**J**
			**RUC**	**RUC**
			**RH**	**RH**
9	HYKSO	F	**J**	**J**
			**C**	**C**
			**RH**	**RH**
			**LH**	**LH**
			**LUC**	**LH**
			**RUC**	**C**
			**J**	**J**
			**C**	**C**
			**C**	**C**
			**LH**	**LH**
			**RH**	**C**
			**LUC**	**LH**
			**RUC**	**C**
			**J**	**J**
			**LUC**	**LH**
			**RH**	**C**
			**LH**	**J**
			**RUC**	**RH**
			**J**	**J**
			**C**	**C**
			**LH**	**J**
			**C**	**C**
			**J**	**J**

			RUC	C
			LH	J
			RUC	C
			J	J
			RH	RH
			LH	LH
			RH	C
			LH	LH
			RUC	C
			LUC	LUC
			RUC	C
			LUC	LH
			C	C
			LH	LH
			C	C
			RUC	C
			LUC	LH
			J	J
			RH	C
			RUC	0
			LH	LH
			RH	C
			RUC	RH
			C	C
			J	J
			C	C
			LUC	LUC
			LH	LH
			J	J
			RUC	RUC
			RH	RH
9	CORNER	F		
			J	J
			C	C
			RH	C
			LH	LH
			LUC	LUC
			RUC	C
			J	J
			C	C
			C	C
			LH	LH

			RH	C
			LUC	LH
			RUC	C
			J	J
			LUC	LUC
			RH	RH
			LH	LH
			RUC	C
			J	J
			C	C
			LH	LH
			C	C
			J	J
			RUC	RH
			LUC	LUC
			RH	RH
			J	J
			RH	C
			LH	LH
			RH	C
			LH	J
			RUC	RUC
			LUC	LH
			RUC	C
			LUC	LUC
			C	C
			LH	J
			C	C
			RH	C
			RUC	RUC
			J	J
			RH	RH
			RUC	C
			LH	LH
			RH	RH
			RUC	C
			C	C
			J	J
			C	C
			LUC	LUC
			LH	LH
			J	J

			**RUC**	**C**
			**RH**	**C**
10	HYKSO	A	**J**	**J**
			**C**	**C**
			**RH**	**0**
			**LH**	**0**
			**LUC**	**0**
			**RUC**	**0**
			**J**	**0**
			**C**	**C**
			**C**	**C**
			**LH**	**0**
			**RH**	**0**
			**LUC**	**0**
			**RUC**	**RUC**
			**J**	**J**
			**LUC**	**0**
			**RH**	**RH**
			**LH**	**LH**
			**RUC**	**RUC**
			**J**	**J**
			**C**	**C**
			**LH**	**0**
			**C**	**C**
			**J**	**J**
			**RH**	**RH**
			**LUC**	**0**
			**RH**	**0**
			**J**	**J**
			**RH**	**RH**
			**LH**	**0**
			**RH**	**0**
			**LH**	**0**
			**RUC**	**RUC**
			**LUC**	**0**
			**RUC**	**RUC**
			**LUC**	**0**
			**C**	**C**
			**LH**	**LH**
			**C**	**C**
			**RUC**	**RUC**
			**LUC**	**LUC**

			**J**	**J**
			**RH**	**C**
			**RUC**	**RUC**
			**LH**	**LH**
			**RH**	**RH**
			**RUC**	**0**
			**C**	**C**
			**J**	**J**
			**C**	**C**
			**LUC**	**LUC**
			**LH**	**LH**
			**C**	**C**
			**RUC**	**RUC**
			**RH**	**RH**
10	CORNER	A	**J**	**J**
			**C**	**C**
			**RH**	**RH**
			**LH**	**LH**
			**LUR**	**LUC**
			**RUC**	**RUC**
			**J**	**J**
			**C**	**C**
			**C**	**C**
			**LH**	**LH**
			**RH**	**RH**
			**LUC**	**LH**
			**RUC**	**RUC**
			**J**	**J**
			**LUC**	**LUC**
			**RH**	**RH**
			**LH**	**LH**
			**RUC**	**RUC**
			**J**	**J**
			**C**	**C**
			**LH**	**LH**
			**C**	**C**
			**J**	**J**
			**RUC**	**RUC**
			**LUC**	**LH**
			**RH**	**RH**
			**J**	**J**
			**RH**	**RH**

			LH	LH
			RH	RH
			LH	LH
			RUC	RUC
			LUC	LUC
			RUC	RUC
			LUC	LUC
			C	C
			LH	LH
			C	C
			RUC	RUC
			LUC	LUC
			J	J
			RH	RH
			RUC	RUC
			LH	J
			RH	RH
			RUC	RUC
			C	C
			J	J
			C	C
			LUC	LH
			LH	LH
			J	J
			RUC	RUC
			RH	RH
11	HYKSO	A	J	J
			C	C
			RH	RH
			LH	LH
			LUC	LUC
			RUC	RUC
			J	J
			C	C
			C	C
			LH	J
			RH	RUC
			LUC	LUC
			RUC	RUC
			J	J
			LUC	LUC
			RH	RH

			LH	LUC
			RUC	RUC
			J	J
			C	C
			LH	LUC
			C	C
			J	J
			RUC	RUC
			LUC	LUC
			RH	RH
			J	J
			RH	RH
			LH	LH
			RH	RH
			LH	LH
			RUC	RUC
			LUC	LUC
			RUC	RH
			LUC	LUC
			C	C
			LH	LH
			C	C
			RUC	RUC
			LUC	LUC
			J	J
			RH	RH
			RUC	RH
			LH	LH
			RH	RH
			RUC	RUC
			C	C
			J	J
			C	C
			LUC	LUC
			LH	LUC
			J	J
			RUC	RUC
			RH	RH
11	CORNER	A	J	J
			C	C
			RH	C
			LH	J

			LUC	LUC
			RUC	RUC
			J	J
			C	C
			C	C
			LH	J
			RH	C
			LUC	LH
			RUC	RUC
			J	J
			LUC	LUC
			RH	RUC
			LH	LH
			RUC	RUC
			J	J
			C	C
			LH	LH
			C	C
			J	J
			RUC	RUC
			LUC	LUC
			RH	C
			J	J
			RH	RH
			LH	LH
			RH	RH
			LH	LH
			RUC	RUC
			LUC	LUC
			RUC	LUC
			LUC	RUC
			C	C
			LH	C
			C	LUC
			RUC	RUC
			LUC	LUC
			J	J
			RH	C
			RUC	RUC
			LH	LH
			RH	RH
			RUC	RUC

			C	C
			J	J
			C	C
			LUC	LUC
			LH	LH
			J	J
			RUC	RUC
			RH	C
12	HYKSO	A	J	J
			C	C
			RH	J
			LH	0
			LUC	0
			RUC	0
			J	J
			C	C
			C	C
			LH	LH
			RH	0
			LUC	LUC
			RUC	RUC
			J	J
			LUC	LUC
			RH	RH
			LH	0
			RUC	RUC
			J	J
			C	C
			LH	J
			C	C
			J	J
			RUC	RUC
			LUC	LH
			RH	RH
			J	J
			RH	0
			LH	J
			RH	0
			LH	0
			RUC	RUC
			LUC	0
			RUC	RUC

			LUC	0
			C	C
			LH	0
			C	C
			RUC	RUC
			LUC	0
			J	J
			RH	0
			RUC	RUC
			LH	LUC
			RH	RH
			RUC	RUC
			C	C
			J	J
			C	C
			LUC	LH
			LH	0
			J	J
			RUC	RUC
			RH	RH
12	CORNER	A	J	J
			C	C
			RH	C
			LH	J
			LUC	LUC
			RUC	RUC
			J	J
			C	C
			C	C
			LH	J
			RH	C
			LUC	0
			RUC	RUC
			J	J
			LUC	LUC
			RH	RH
			LH	LH
			RUC	0
			J	J
			C	C
			LH	LH
			C	C

			J	J
			RUC	RUC
			LUC	LUC
			RH	RH
			J	J
			RH	RH
			LH	LH
			RH	RH
			LH	J
			RUC	RUC
			LUC	LUC
			RUC	RUC
			LUC	LUC
			C	C
			LH	LH
			C	C
			RUC	0
			LUC	LUC
			J	J
			RH	C
			RUC	RUC
			LH	LH
			RH	C
			RUC	RUC
			C	C
			J	J
			C	C
			LUC	LUC
			LH	LH
			J	J
			RUC	RUC
			RH	RH
13	HYKSO	F	J	J
			C	C
			RH	RH
			LH	LH
			LUC	LUC
			RUC	RUC
			J	J
			C	C
			C	C
			LH	LH

			RH	RH
			LUC	LUC
			RUC	RUC
			J	J
			LUC	0
			RH	RH
			LH	LH
			RUC	RUC
			J	J
			C	C
			LH	LH
			C	C
			J	J
			RUC	RUC
			LUC	0
			RH	RH
			J	J
			RH	RH
			LH	LH
			RH	C
			LH	LH
			RUC	RUC
			LUC	LUC
			RUC	RUC
			LUC	LUC
			J	J
			LH	LH
			J	J
			RUC	RUC
			LUC	LUC
			J	J
			RH	RH
			RUC	RUC
			LH	LH
			RH	RH
			RUC	RUC
			C	C
			J	J
			C	C
			LUC	LUC
			LH	LH
			J	J

			RUC	RUC
			RH	RH
13	CORNER	F	J	J
			C	C
			RH	RH
			LH	LH
			LUC	LUC
			RUC	RUC
			J	J
			C	C
			J	J
			RH	C
			RH	RH
			LUC	LUC
			RUC	RUC
			J	J
			LUC	LUC
			RH	RH
			LH	LH
			RUC	RUC
			J	J
			C	C
			LH	LH
			C	C
			J	J
			RUC	RUC
			LUC	LUC
			RH	RH
			J	J
			RH	RH
			LH	LH
			RH	RH
			LH	LH
			RUC	RUC
			LUC	LUC
			RUC	RUC
			LUC	LUC
			C	C
			LH	LH
			C	C
			RUC	RUC
			LUC	LUC

			**J**	**J**
			**RH**	**RH**
			**RUC**	**RUC**
			**LH**	**LH**
			**RH**	**RH**
			**RUC**	**RUC**
			**C**	**C**
			**J**	**J**
			**C**	**C**
			**LUC**	**LUC**
			**LH**	**LH**
			**J**	**J**
			**RUC**	**RUC**
			**RH**	**RH**
14	HYKSO	F	**J**	**J**
			**0**	**J**
			**C**	**C**
			**0**	**J**
			**RH**	**RH**
			**LH**	**LUC**
			**LUC**	**0**
			**0**	**J**
			**RUC**	**RUC**
			**J**	**J**
			**C**	**C**
			**C**	**C**
			**LH**	**LH**
			**RH**	**RH**
			**LUC**	**LH**
			**RUC**	**RUC**
			**J**	**J**
			**LUC**	**LUC**
			**RH**	**RUC**
			**LH**	**LH**
			**RUC**	**RH**
			**J**	**J**
			**C**	**C**
			**LH**	**LH**
			**C**	**C**
			**J**	**J**
			**RUC**	**RUC**
			**LUC**	**LH**

			RH	RH
			J	J
			RH	RH
			LH	LH
			RH	RH
			LH	LH
			RUC	RH
			LUC	LH
			RUC	RUC
			LUC	LUC
			C	C
			LH	LH
			C	C
			RUC	RUC
			LUC	LH
			J	J
			RH	RH
			RUC	RH
			LH	LH
			RH	RH
			0	J
			RUC	RUC
			C	C
			J	J
			C	C
			LUC	LUC
			LH	0
			J	J
			0	LH
			RUC	RUC
			0	J
			RH	C
14	CORNER	F	J	J
			C	C
			RH	RH
			LH	LH
			0	RUC
			LUC	LH
			0	RUC
			0	J
			RUC	RUC
			J	J

			C	C
			C	C
			LH	LH
			RH	RH
			LUC	LUC
			0	J
			RUC	RUC
			J	J
			LUC	LH
			0	RH
			RH	RUC
			LH	LH
			RUC	RUC
			J	J
			C	C
			LH	J
			C	C
			J	J
			RUC	C
			LUC	LH
			RH	RUC
			J	J
			RH	RH
			0	RUC
			LH	LH
			RH	RH
			LH	LH
			0	RUC
			RUC	C
			LUC	J
			RUC	RUC
			LUC	LUC
			C	0
			LH	LH
			RUC	RUC
			RUC	RUC
			LUC	J
			0	C
			0	C
			J	J
			RH	RH
			RUC	RUC

			**LH**	**LH**
			**RH**	**C**
			**0**	**J**
			**RUC**	**RUC**
			**C**	**C**
			**J**	**J**
			**0**	**LH**
			**C**	**RUC**
			**0**	**C**
			**LUC**	**LH**
			**LH**	**LH**
			**J**	**J**
			**0**	**C**
			**0**	**LUC**
			**RUC**	**RUC**
			**0**	**C**
			**RH**	**C**
15	HYKSO	A	**J**	**J**
			**C**	**C**
			**RH**	**0**
			**LH**	**0**
			**LUC**	**0**
			**RUC**	**0**
			**J**	**0**
			**C**	**C**
			**C**	**C**
			**LH**	**LUC**
			**RH**	**RH**
			**LUC**	**LH**
			**RUC**	**0**
			**J**	**J**
			**LUC**	**LUC**
			**RH**	**RH**
			**LH**	**LH**
			**RUC**	**0**
			**J**	**J**
			**C**	**C**
			**LH**	**0**
			**C**	**C**
			**J**	**J**
			**RUC**	**0**
			**LUC**	**LUC**

			RH	RUC
			J	J
			RH	RH
			LH	0
			RH	0
			LH	0
			RUC	RUC
			J	J
			RUC	RUC
			LUC	0
			C	C
			LH	LH
			C	C
			RUC	RUC
			LUC	LH
			J	J
			RH	RH
			RUC	RUC
			LH	LUC
			RH	RH
			RUC	RUC
			C	C
			J	J
			C	C
			LUC	LUC
			LH	LH
			J	J
			RUC	RUC
			RH	RUC
15	CORNER	A	J	J
			C	C
			RH	RH
			LH	LH
			LUC	LUC
			RUC	RUC
			J	J
			C	C
			C	C
			LH	LUC
			RH	RUC
			LUC	LUC
			RUC	RUC

			J	J
			LUC	LUC
			RH	RUC
			LH	LH
			RUC	RUC
			J	J
			C	C
			LH	LUC
			C	C
			J	J
			RUC	RUC
			LUC	LUC
			RH	RUC
			J	J
			RH	RH
			LH	LUC
			RH	RH
			LH	LH
			RUC	RUC
			LUC	LUC
			RUC	RUC
			LUC	LUC
			C	C
			LH	LH
			C	C
			RUC	RUC
			LUC	LUC
			J	J
			RH	RUC
			RUC	RUC
			LUC	LUC
			RH	RH
			RUC	RUC
			C	C
			J	J
			C	C
			LUC	LUC
			LH	LH
			J	J
			RUC	RUC
			RH	RH
16	HYKSO	F	J	J

			C	C
			RH	C
			LH	LH
			LUC	LH
			RUC	RUC
			J	J
			C	C
			C	C
			LH	LH
			RH	C
			LUC	LH
			RUC	RUC
			J	J
			LUC	LUC
			RH	C
			LH	LH
			RUC	RH
			J	J
			C	C
			LH	J
			C	C
			J	J
			RUC	RH
			LUC	LUC
			RH	C
			J	J
			RH	C
			LH	LH
			RH	C
			LH	LH
			RUC	RH
			LUC	LUC
			RUC	RUC
			LUC	LUC
			C	C
			LH	LH
			C	C
			RUC	RUC
			LUC	LUC
			J	J
			RH	C
			RUC	RH

			**LH**	**LH**
			**RH**	**C**
			**RUC**	**RUC**
			**C**	**C**
			**J**	**J**
			**C**	**C**
			**LUC**	**LUC**
			**LH**	**LH**
			**J**	**J**
			**RUC**	**RH**
			**RH**	**C**
16	CORNER	F	**J**	**J**
			**C**	**C**
			**RH**	**C**
			**LH**	**J**
			**LUC**	**LUC**
			**RUC**	**RUC**
			**J**	**C**
			**C**	**J**
			**C**	**C**
			**LH**	**J**
			**RH**	**LUC**
			**LUC**	**C**
			**RUC**	**C**
			**J**	**J**
			**LUC**	**LUC**
			**RH**	**C**
			**LH**	**RUC**
			**RUC**	**J**
			**J**	**J**
			**C**	**C**
			**LH**	**J**
			**C**	**C**
			**J**	**C**
			**RUC**	**J**
			**LUC**	**LUC**
			**RH**	**C**
			**J**	**J**
			**RH**	**C**
			**LH**	**LH**
			**RH**	**C**
			**LH**	**J**

			RUC	RUC
			LUC	LUC
			RUC	RUC
			LUC	LUC
			C	C
			LH	LH
			C	C
			RUC	LUC
			LUC	C
			J	J
			RH	C
			RUC	C
			LH	LH
			RH	C
			RUC	C
			C	J
			J	C
			C	C
			LUC	LUC
			LH	J
			J	J
			RUC	RUC
			RH	C
17	HYKSO	F	J	J
			C	C
			RH	C
			LH	LH
			LUC	LUC
			RUC	RH
			J	J
			C	C
			C	C
			LH	J
			RH	C
			LUC	LH
			RUC	RH
			J	J
			LUC	J
			RH	C
			LH	J
			RUC	RUC
			J	J

			C	C
			LH	LH
			C	RH
			J	J
			RUC	0
			LUC	J
			RH	C
			J	J
			RH	C
			LH	LH
			RH	C
			LH	J
			RUC	RUC
			LUC	LH
			RUC	RUC
			LUC	J
			C	C
			LH	LH
			C	C
			RUC	RUC
			LUC	LH
			J	J
			RH	C
			RUC	RH
			LH	J
			RH	C
			LUC	LH
			C	C
			J	J
			C	C
			LUC	LUC
			LH	LH
			J	0
			RUC	RUC
			RH	C
17	CORNER	F	J	J
			C	C
			RH	C
			LH	LH
			LUC	LUC
			RUC	RUC
			J	J

			C	C
			C	C
			LH	LH
			RH	C
			LUC	LH
			RUC	0
			J	J
			LUC	LUC
			RH	RH
			LH	LH
			RUC	RUC
			J	J
			C	C
			LH	LH
			C	C
			J	RUC
			RUC	J
			LUC	C
			RH	LH
			J	J
			RH	C
			LH	LH
			RH	C
			LH	J
			RUC	RUC
			LUC	LUC
			RUC	RUC
			LUC	LUC
			C	C
			LH	LH
			C	C
			RUC	LUC
			LUC	RUC
			J	J
			RH	C
			RUC	RUC
			LH	LH
			RH	C
			RUC	RUC
			C	C
			J	J
			C	C

			**LUC**	**LUC**
			**LH**	**LH**
			**J**	**J**
			**RUC**	**RUC**
			**RH**	**C**
18	HYKSO	A	**J**	**J**
			**C**	**C**
			**RH**	**RH**
			**LH**	**J**
			**LUC**	**LUC**
			**RUC**	**RUC**
			**J**	**J**
			**C**	**C**
			**C**	**C**
			**LH**	**J**
			**RH**	**RH**
			**LUC**	**LH**
			**RUC**	**RUC**
			**J**	**J**
			**LUC**	**J**
			**RH**	**RH**
			**LH**	**LH**
			**RUC**	**C**
			**J**	**J**
			**C**	**C**
			**LH**	**J**
			**C**	**C**
			**J**	**J**
			**RH**	**RH**
			**LUC**	**J**
			**RH**	**RH**
			**J**	**J**
			**RH**	**RH**
			**LH**	**J**
			**RH**	**RUC**
			**LH**	**LH**
			**RUC**	**RUC**
			**LUC**	**LH**
			**RUC**	**RUC**
			**LUC**	**LH**
			**C**	**RH**
			**LH**	**LH**

			**C**	**C**
			**RUC**	**C**
			**LUC**	**LUC**
			**J**	**J**
			**RH**	**RH**
			**RUC**	**RUC**
			**LH**	**LUC**
			**LH**	**LH**
			**RUC**	**RUC**
			**C**	**C**
			**J**	**J**
			**C**	**C**
			**LUC**	**LUC**
			**RH**	**RH**
			**J**	**J**
			**RUC**	**RUC**
			**RH**	**RH**
18	CORNER	A	**J**	**J**
			**C**	**C**
			**RH**	**RH**
			**LH**	**J**
			**LUC**	**J**
			**RUC**	**RUC**
			**J**	**J**
			**C**	**C**
			**C**	**C**
			**LH**	**J**
			**RH**	**RUC**
			**LUC**	**J**
			**RUC**	**C**
			**J**	**J**
			**LUC**	**J**
			**RH**	**RUC**
			**LH**	**J**
			**RUC**	**C**
			**J**	**0**
			**C**	**C**
			**LH**	**J**
			**C**	**C**
			**J**	**J**
			**RUC**	**C**
			**LUC**	**J**

			RH	RUC
			J	J
			RH	C
			LH	LUC
			RH	RUC
			LH	J
			RUC	C
			LUC	J
			RUC	C
			LUC	J
			C	C
			LH	J
			C	C
			RUC	RUC
			LUC	J
			J	J
			RH	RH
			RUC	C
			LH	LUC
			RH	RUC
			RUC	C
			C	C
			J	J
			C	C
			LUC	J
			LH	J
			J	J
			RUC	RUC
			RH	RUC
19	HYKSO	F	J	J
			C	C
			RH	C
			LH	0
			LUC	LUC
			RUC	C
			J	J
			C	C
			C	0
			LH	LH
			RH	C
			LUC	LH
			RUC	RH

			J	J
			LH	J
			RH	C
			LH	J
			RUC	RUC
			J	J
			C	C
			LH	J
			C	RH
			J	J
			RUC	C
			LUC	LH
			RH	RUC
			J	J
			RH	C
			LH	LUC
			RH	C
			LH	J
			RUC	RH
			LUC	LH
			RUC	RH
			LUC	LH
			C	C
			LH	J
			C	C
			RUC	RH
			LUC	LH
			J	J
			LH	C
			RUC	RH
			LH	LH
			RH	C
			LUC	LH
			C	C
			J	J
			C	C
			LH	J
			LUC	LH
			J	J
			RUC	C
			RH	C
19	CORNER	F	J	LH

			C	C
			0	J
			RH	C
			LH	J
			LUC	LUC
			RUC	RUC
			J	LH
			C	C
			C	C
			LH	LH
			RH	RH
			LUC	LUC
			RUC	RUC
			J	LH
			LUC	LUC
			RH	RH
			LH	J
			RUC	RUC
			J	LH
			C	C
			LH	J
			0	J
			C	C
			J	LUC
			RUC	RUC
			LUC	LUC
			RH	RUC
			J	LH
			RH	RH
			LH	LH
			RH	C
			LH	J
			RUC	C
			LUC	LH
			RUC	C
			LUC	LH
			C	C
			LH	J
			C	C
			RUC	C
			LUC	LUC
			J	LH

			RH	C
			RUC	RUC
			LH	LH
			RH	RH
			0	J
			RUC	RUC
			0	J
			C	C
			J	LUC
			0	J
			C	C
			LUC	LUC
			LH	J
			J	LH
			RUC	C
			RH	RH
20	HYKSO	F	J	J
			C	C
			RH	RH
			LH	LH
			LUC	LUC
			RUC	RUC
			J	J
			C	C
			C	C
			LH	LH
			RH	RH
			LUC	LUC
			RUC	RUC
			J	J
			LUC	LUC
			RH	RH
			LH	LH
			RUC	RUC
			J	J
			C	C
			LH	LUC
			C	C
			J	J
			RUC	RUC
			LUC	LUC
			RH	RH

			J	J
			RH	RH
			LH	LH
			RH	RH
			LH	LH
			RUC	RUC
			LUC	LH
			RUC	RUC
			LUC	LUC
			C	C
			LH	LH
			C	C
			RUC	RUC
			LUC	LH
			J	J
			RH	RH
			RUC	RUC
			LH	LH
			RH	RUC
			LUC	LUC
			C	C
			J	J
			C	C
			LUC	LUC
			LH	LH
			J	J
			RUC	RUC
			RH	RH
20	CORNER	F	J	J
			C	C
			RH	C
			LH	LH
			LUC	LUC
			RUC	RUC
			J	J
			C	C
			C	C
			LH	LUC
			RH	C
			LUC	LUC
			RUC	RUC
			J	J

			LUC	LUC
			RH	RUC
			LH	LH
			RUC	RUC
			J	C
			C	J
			LH	LH
			C	C
			J	RUC
			RUC	J
			LUC	LUC
			RH	RUC
			J	J
			RH	RH
			LH	LH
			RH	C
			LH	LH
			RUC	RUC
			LH	LH
			RUC	RUC
			LUC	LUC
			C	C
			LH	LH
			C	C
			RUC	RUC
			LUC	LUC
			J	J
			RH	C
			RUC	RUC
			LH	LH
			RH	RUC
			RUC	RUC
			C	C
			J	J
			C	C
			LUC	LUC
			LH	LH
			J	J
			RUC	RUC
			RH	RH
21	HYKSO	F	J	J
			C	C

			RH	RH
			LH	J
			LUC	LUC
			RUC	RUC
			J	J
			C	C
			C	C
			LH	0
			RH	RH
			LUC	0
			RUC	RUC
			J	J
			LUC	LUC
			RH	0
			LH	LH
			RUC	RUC
			J	J
			C	C
			LH	LH
			C	C
			J	J
			0	0
			LUC	LUC
			RH	RH
			J	J
			RH	RH
			LH	LH
			RH	0
			LH	LH
			RUC	RUC
			LH	0
			RUC	RUC
			LUC	J
			C	C
			LH	LH
			C	C
			RUC	RUC
			LUC	LH
			J	J
			RH	RH
			RUC	RUC
			LH	LH

			RH	RH
			RUC	RUC
			J	J
			C	C
			C	C
			LUC	LUC
			LH	J
			J	J
			RUC	RUC
			RH	C
21	CORNER	F	J	J
			C	C
			RH	RH
			LH	LH
			LUC	LUC
			RUC	RUC
			J	0
			C	C
			C	C
			0	0
			RH	RH
			LUC	LUC
			RH	RH
			J	J
			LUC	LUC
			RH	RH
			LH	LH
			RUC	RUC
			J	J
			C	C
			LH	LH
			C	C
			J	J
			RUC	RUC
			LUC	LUC
			RH	RH
			J	0
			RH	RH
			LH	LH
			RH	RH
			LH	LH
			RUC	RUC

			LUC	LH
			RUC	RUC
			LUC	LUC
			C	C
			LH	LH
			C	C
			RUC	RUC
			LUC	LUC
			J	J
			RH	RH
			RUC	RUC
			LH	LH
			RH	RH
			RUC	RUC
			J	J
			C	C
			C	C
			LUC	LUC
			LH	LH
			J	J
			RUC	RUC
			RH	RH
